# Comparative temporal and dose-dependent morphological and transcriptional uterine effects elicited by tamoxifen and ethynylestradiol in immature, ovariectomized mice

**DOI:** 10.1186/1471-2164-8-151

**Published:** 2007-06-07

**Authors:** Cora J Fong, Lyle D Burgoon, Kurt J Williams, Agnes L Forgacs, Timothy R Zacharewski

**Affiliations:** 1 Department of Biochemistry & Molecular Biology, Michigan State University, East Lansing, MI, 48824, USA; 2 National Food Safety & Toxicology Center, Michigan State University, East Lansing, MI, 48824, USA; 3 Center for Integrative Toxicology, Michigan State University, East Lansing, MI, 48824, USA; 4 Department of Pathobiology & Diagnostic Investigation, Michigan State University, East Lansing, MI, 48824, USA

## Abstract

**Background:**

Uterine temporal and dose-dependent histopathologic, morphometric and gene expression responses to the selective estrogen receptor modulator tamoxifen (TAM) were comprehensively examined to further elucidate its estrogen receptor-mediated effects. These results were systematically compared to the effects elicited by the potent estrogen receptor ligand 17α-ethynylestradiol (EE) to identify pathways similarly and uniquely modified by each compound.

**Results:**

Three daily doses of 100 μg/kg TAM elicited a dose-dependent increase in uterine wet weight (UWW) in immature, ovariectomized C57BL/6 mice at 72 hrs with concurrent increases in luminal epithelial cell height (LECH), luminal circumference and glandular epithelial tubule number. Significant UWW and LECH increases were detected at 24 hrs after a single dose of 100 μg/kg TAM. cDNA microarray analysis identified 2235 differentially expressed genes following a single dose of 100 μg/kg TAM at 2, 4, 8, 12, 18 and 24 hrs, and at 72 hrs after three daily doses (3 × 24 hrs). Functional annotation of differentially expressed genes was associated with cell growth and proliferation, cytoskeletal organization, extracellular matrix modification, nucleotide synthesis, DNA replication, protein synthesis and turnover, lipid metabolism, glycolysis and immunological responses as is expected from the uterotrophic response. Comparative analysis of TAM and EE treatments identified 1209 common, differentially expressed genes, the majority of which exhibited similar profiles despite a temporal delay in TAM elicited responses. However, several conserved and treatment specific responses were identified that are consistent with proliferation (Fos, Cdkn1a, Anapc1), and water imbibition (Slc30a3, Slc30a5) responses elicited by EE.

**Conclusion:**

Overall, TAM and EE share similar gene expression profiles. However, TAM responses exhibit lower efficacy, while responses unique to EE are consistent with the physiological differences elicited between compounds.

## Background

Tamoxifen (TAM) treatment is an adjuvant therapy prescribed for estrogen receptor positive breast cancers. TAM and its metabolites, 4-hydroxytamoxifen (4OH-TAM), *N*-desmethyltamoxifen (DMT) and 4-OH-*N*-desmethyltamoxifen (endoxifen), exhibit antiestrogenic activities by competitively inhibiting the binding of potent agonists to the estrogen receptor (ER) thus antagonizing their proliferative effects [1-4]. Despite the high therapeutic index of TAM for breast cancer, there are concerns regarding the increased occurrence of uterine cancer as early as 2 years after initiating treatment [5]. Although there is no direct evidence that it initiates or promotes uterine cancer, TAM exhibits partial ER-agonist activity by inducing uterotrophy in immature and ovariectomized rodents [6,7]. Consequently, a more comprehensive comparison to full agonists is warranted to further elucidate the uterine gene expression effects responsible for its partial agonist activity.

TAM is classified as a selective estrogen receptor modulator (SERM) as a result of its differential effects in breast and uterine tissues [8]. A number of factors influence the specificity and efficacy of SERM-bound, ER-mediated gene expression, and the subsequent physiological effects. This includes differences in tissue-specific ER isoform expression levels, ligand-induced ER topology, chromatin structure, and coactivator expression and distribution [9,10], thus making the ER an ideal target for drug discovery and development. For example, raloxifene, a second-generation SERM, has been approved for osteoporosis and studies also support its use for breast cancer [11].

The uterotrophic assay is a well established method to evaluate the estrogenicity of a compound as measured by ER-mediated increases in uterine wet weight making it an ideal model for comparing 17α-ethynylestradiol (EE) and TAM elicited effects [12]. The uterotrophic response also provides well characterized phenotypic hallmarks that facilitate the interpretation of gene expression changes and their function. Early studies have shown that TAM elicits a weaker uterotrophic response than 17β-estradiol (E2) in an immature rodent model [13], however, the mechanisms for its partial agonist activity are not well understood.

Genome-wide expression analysis, phenotypically anchored to tissue level effects, provides a comprehensive strategy to identify differential gene expression important in the ER-induction of uterine wet weight. In this report, we extend previous studies examining ER-mediated induction of uterine wet weight [14-16] by identifying conserved and divergent uterine tissue and gene expression responses elicited by TAM when compared to EE, an orally active full agonist that mimics the effects of E2 [17]. Comparative analysis found conserved gene expression responses that exhibited lower efficacy, consistent with the weak agonist activity of TAM, as well as divergent responses unique to EE that partially explain the lack of TAM-induced water imbibition.

## Results

### Uterine weight

**Figure 1:**
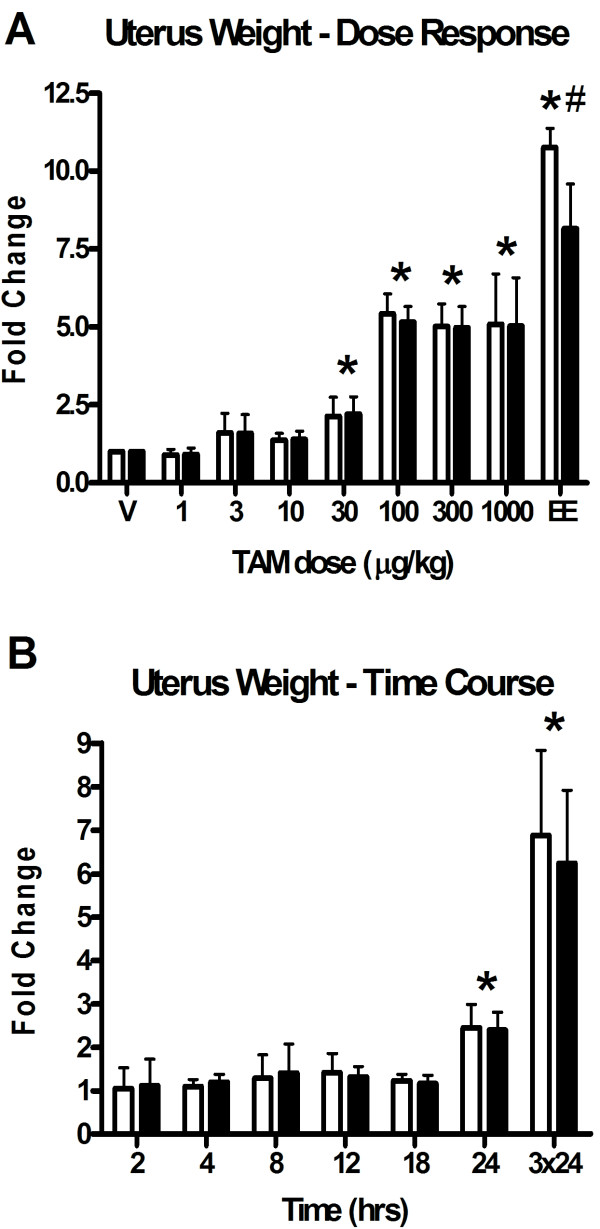
**Tamoxifen-induced dose dependent and temporal changes in uterine weight**. Graphs illustrate fold-change increases in uterine wet (open) and blotted (solid) weight. A) Tamoxifen elicits a dose dependent uterotrophic response (EC_50 _= 33.7 μg/kg) and achieves maximal induction of approximately 5-fold following three daily doses (3 × 24 hrs) of 100 μg/kg TAM. Significant increases (p < 0.05, n = 5) are denoted by an asterisk (*). In contrast, 100 μg/kg EE (positive control) maximally induced uterine wet weight 11-fold (*, p < 0.05, n = 5) with significant water imbibition (#; p < 0.05, n = 3), while TAM only achieved 50% uterotrophic efficacy and no water imbibition. B) A single dose of 100 μg/kg TAM significantly increased uterine wet weight as early as 24 hrs after administration. No significant water imbibition was observed at any time point.

Increases in uterine wet weight (UWW) in rodents after three daily subcutaneous doses of TAM is well documented [18,19]. Dose-dependent increases in uterine weight (EC_50 _= 33.7 μg/kg) were observed following three consecutive daily oral treatments of TAM (Figure 1A), however induction plateaued at 5-fold, compared to 11-fold with an equivalent dose of 100 μg/kg 17α-ethynylestradiol (EE) [16]. Comparison of wet and blotted uterine weights indicated no significant water imbibition in TAM-treated uteri. However, blotted EE-treated uteri were larger, consistent with past reports that TAM induces a less efficacious uterotrophic effect [20]. In order to establish a temporal profile, the uterotrophic effects of 100 μg/kg TAM were also investigated at 2, 4, 8, 12, 18, 24 and 3 × 24 hrs. A significant 2.5-fold increase was observed at 24 hrs after a single 100 μg/kg TAM dose (Figure 1B) which was delayed compared to the significant increase seen with 100 μg/kg EE at 18 hrs [16].

### Morphometric analysis and histopathology

**Figure 2:**
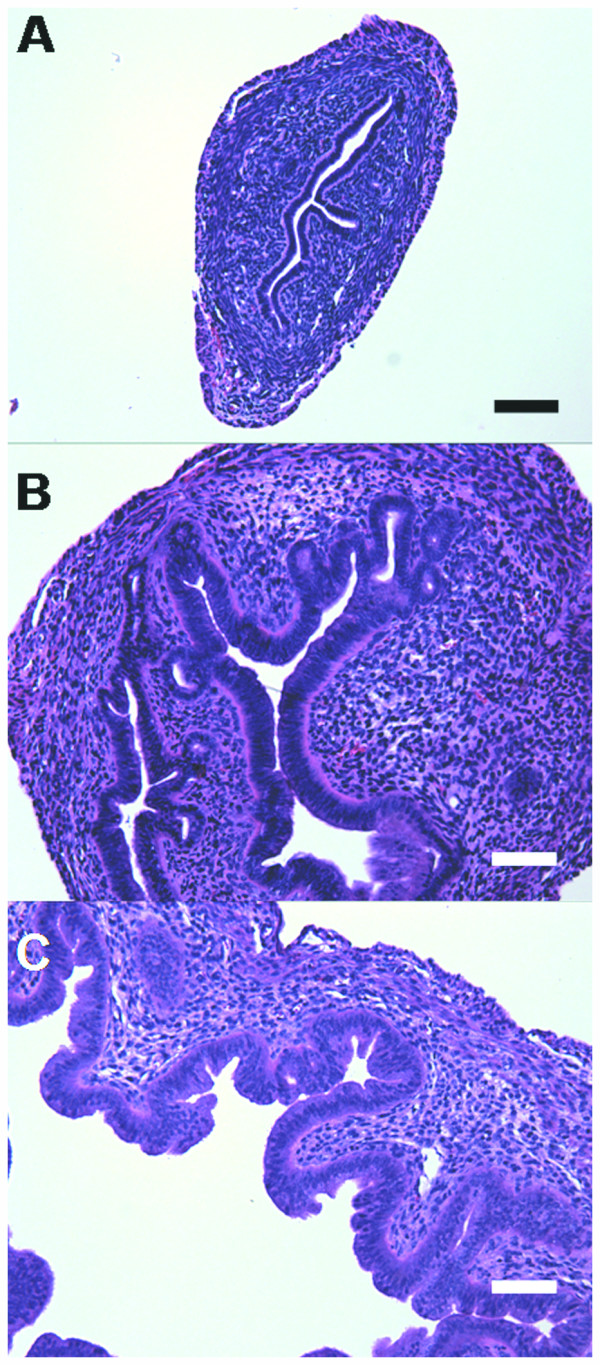
**Uterine histology**. Hematoxylin and eosin stained sections of uterine tissue at 100× magnification after three daily doses of A) sesame oil, B) 1 mg/kg TAM and C) 100 μg/kg EE. TAM and EE treatment induced increases in luminal epithelial cell height. Luminal circumference is increased to a greater degree by EE than TAM. Bars represent 20 μm.

Luminal epithelial cell height (LECH), luminal circumference and number of endometrial glands are hallmarks of estrogen action in the rodent which correlate with UWW induction [21]. Significant dose-dependent increases in LECH and luminal circumference were initially detected at 30 μg/kg TAM (Table 1A). Interestingly, LECH was not significantly different between 100 μg/kg EE and TAM, although the luminal circumference of EE uteri was greater with more pronounced invagination of the luminal glandular epithelium (Figure 2). There was also mild to moderate hypertrophy in the stromal nuclei at 10 μg/kg TAM with moderate epithelial hypertrophy and hyperplasia at 30 μg/kg TAM, which was marked at higher doses. Mild edema was noted for all samples beginning at 100 μg/kg TAM. Marked to severe stromal nuclei hypertrophy and epithelial hypertrophy and hyperplasia, all with mild edema, was observed at 100 μg/kg EE. Mild to moderate stromal edema was observed as early as 12 hrs following after a single 100 μg/kg TAM dose, while increased UWW and LECH were not significant until 24 hrs (Table 1B). No significant increase in luminal circumference was observed in the first 24 hrs after treatment.

**Table 1:** TAM- and EE-induced uterine morphometric changes

A)			
Dose Response (3 × 24 hr)			

TAM Dose (μg/kg)	Luminal Epithelial Cell Height (μm)	Luminal Circumference (mm)	Avg. Number of Glandular Tubules

0	8.75 ± 0.86	0.77 ± 0.14	1
1	8.99 ± 1.00	0.72 ± 0.12	0
3	10.91 ± 2.97	1.17 ± 0.41	1
10	10.73 ± 1.15	1.17 ± 0.29	3
30	15.12 ± 1.55*	1.87 ± 0.26*	5*
100	24.58 ± 2.79*	3.60 ± 0.27*	10*
300	27.08 ± 3.79*	2.68 ± 1.19*	5*
1000	31.30 ± 2.25*	3.05 ± 0.73*	5*
100 EE	28.94 ± 3.35*	+++^a^	4

B)			

Time Course (100 μg/kg)			

Time (hrs)	Luminal Epithelial Cell Height (μm)	Luminal Circumference (mm)	

2	9.98 ± 1.68	0.79 ± 0.19	
4	8.61 ± 1.58	0.80 ± 0.06	
8	10.06 ± 2.50	0.96 ± 0.29	
12	9.46 ± 1.28	0.99 ± 0.21	
18	9.18 ± 1.03	1.29 ± 0.42	
24	11.08 ± 1.94*	1.22 ± 0.42	
3 × 24	28.61 ± 7.50*	2.85 ± 1.83*	

* Statistically different from time matched vehicle (p < 0.05)

^a ^Lumen larger than 100× field of view, accurate measurements could not be made at 40× magnification Time course vehicle samples are not significantly different from each other.

Uterine endometrial glands synthesize and secrete fluids in preparation for conceptus, implantation and growth. Significant increases in the number of glands was observed at 30 μg/kg TAM (Table 1A) in the absence of a dose responsive increase, which may be an artifact of histological sampling of the uterine horn. Similarly, EE-treated uteri exhibited an increased number of endometrial glands that was not statistically significant.

### Uterine gene expression changes elicited by tamoxifen

Differentially expressed genes in the dose and time dependent studies were identified based on their empirical Bayes posterior probability of activity [P1(*t*)-value] on a per-gene, per-time point basis. P1(*t*)-values approaching 1.0 indicate a greater likelihood of treatment-related differential gene expression. Using P1(*t*) > 0.999 and |fold change| ≥ 1.5 as selection criteria, a prioritized list of 2941 features, representing 2235 unique Entrez Gene annotated genes, were identified in the temporal study with 55% of the genes exhibiting induction and 45% repression (Additional file 1). Differential expression levels ranged from 14.3-fold repression (tight junction protein 4, *Tjp4*) to 28.1-fold induction (arginase 1, *Arg1*), further demonstrating the responsiveness of the uterus to tamoxifen. Using the same selection criteria (P1(*t*) > 0.999 and |fold change| of ≥ 1.5) at a minimum of three doses, to ensure dose responsiveness, 1630 features, representing 1036 unique Entrez Gene-annotated genes, exhibited dose dependent expression (Additional file 2). Of the 1036 genes exhibiting a dose-dependent response at 3 × 24 hrs and of the 738 differentially expressed genes at 3 × 24 hrs in the time course study, 691 genes (94%) were in common, demonstrating good reproducibility between experiments.

**Figure 3:**
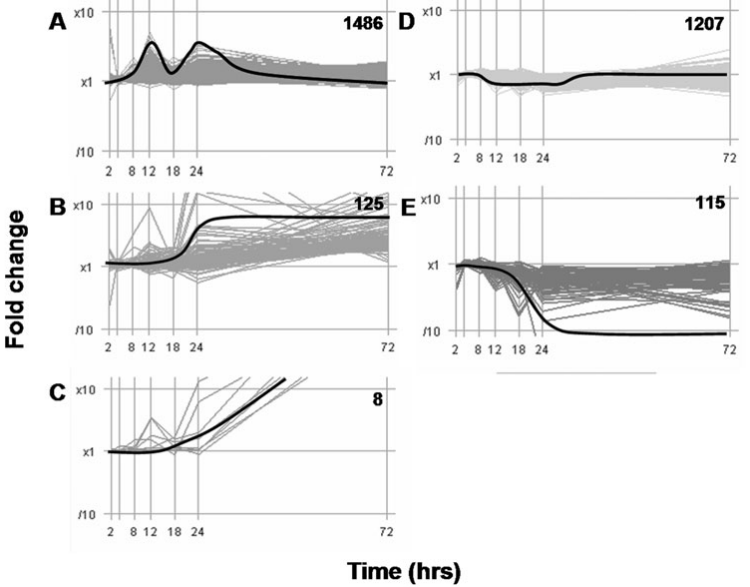
**Tamoxifen-induced temporal gene expression patterns**. Five *k*-means clusters best represent the general temporal patterns for the 2941 features differentially expressed following TAM treatment. Note the 8 hr delay in gene expression response especially in comparison to EE elicited gene expression [16] is speculated to be due to the delayed absorption of TAM. Inset numbers indicate the number of features represented by each cluster. Black pseudolines indicate the general profile represented within each cluster.

Differentially expressed genes were associated with cell growth and proliferation, cytoskeletal organization, extracellular matrix modification, nucleotide synthesis, DNA replication, protein synthesis and turnover, lipid metabolism, glycolysis and immunological responses. The temporal changes in gene expression were best represented using five k-means clusters: A) induced at 12 and 24 hrs, B) induced and sustained from 24 – 72 hrs, C) induced late at 72 hrs, D) repressed between 8 – 24 hrs and E) repressed and sustained from 24 – 72 hrs (Figure 3). The majority of TAM-elicited differential expression occurred after 12 hrs with only 42 features (26 genes) exhibiting differential gene expression between 2 and 8 hrs, in marked contrast to EE studies where significant gene expression changes occurred prior to 8 hrs [15,16,22]. The temporal pattern of differential gene expression correlates with the histology results which indicate a delayed response in comparison to EE.

**Figure 4:**
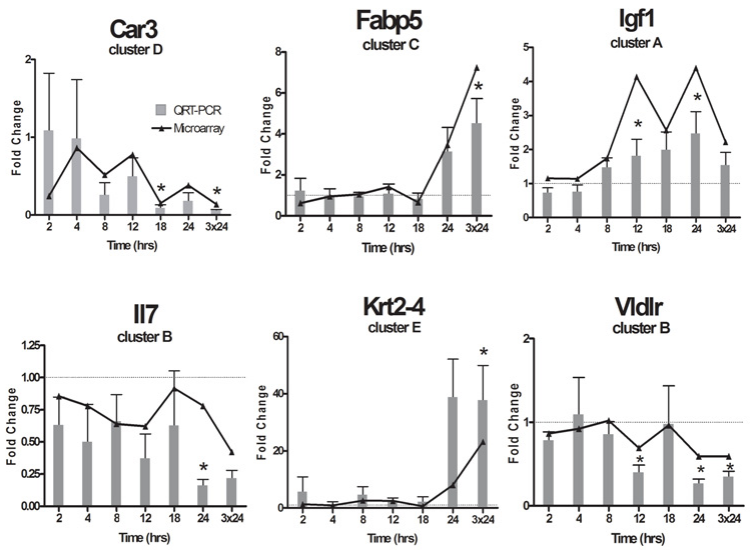
**Quantitative real-time PCR verification of selected TAM-induced genes**. Overall, the microarray results for 14 TAM- and EE-induced genes were verified using QRT-PCR. The verified genes represent various affected pathways and different temporal patterns of expression. Overall, there was good correlation (average ρ = 0.8) between microarray (lines) and QRT-PCR (bars) data. Examples for six of the genes are illustrated. Statistically significant QRT-PCR differences (p < 0.05, n = 4) due to treatment are denoted by an asterisk (*).

Eleven genes, representative of affected pathways and exhibiting different temporal gene expression patterns (i.e. cytoskeletal organization (*Krt2-4*), signal transduction (*Igf1*), immunological responses (*Il7*), acid-base homeostasis (*Car3*) and lipid transport (*Fabp5, Vldlr*)), were verified by QRT-PCR and exhibited good agreement with microarray results. Correlation coefficients ranged from 0.46 to 0.97 (mean = 0.80) (Figure 4).

**Figure 5:**
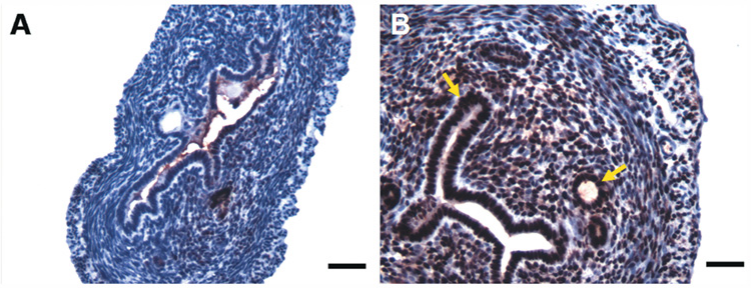
**Immunohistochemical detection of differential Pcna protein levels due to TAM**. Twelve-hour vehicle (A) and TAM (B) treated uteri sections were immunohistochemically stained (NovaRED^®^) with Pcna specific antibodies. Treated samples have darker nuclear staining, indicating greater levels of Pcna protein expression, in agreement with the histological assessment and changes in gene expression associated with cell proliferation. Increased Pcna expression is more pronounced in the luminal and glandular epithelium, and stroma (arrows). Tissues were counter-stained with hematoxylin. Images are representative of four biological replicates. Bars represent 20 μm.

Immunohistochemistry (IHC) was also used to assess and localize PCNA protein expression following TAM treatment (Figure 5). Microarray results indicate a 2.5-fold increase in *Pcna *transcript levels between 12 – 18 hrs after treatment with IHC confirming elevated protein expression in epithelial and stromal cells in 12 hr TAM treated samples when compared to time matched controls.

### Comparison of common temporal TAM and EE gene expression data

**Figure 6:**
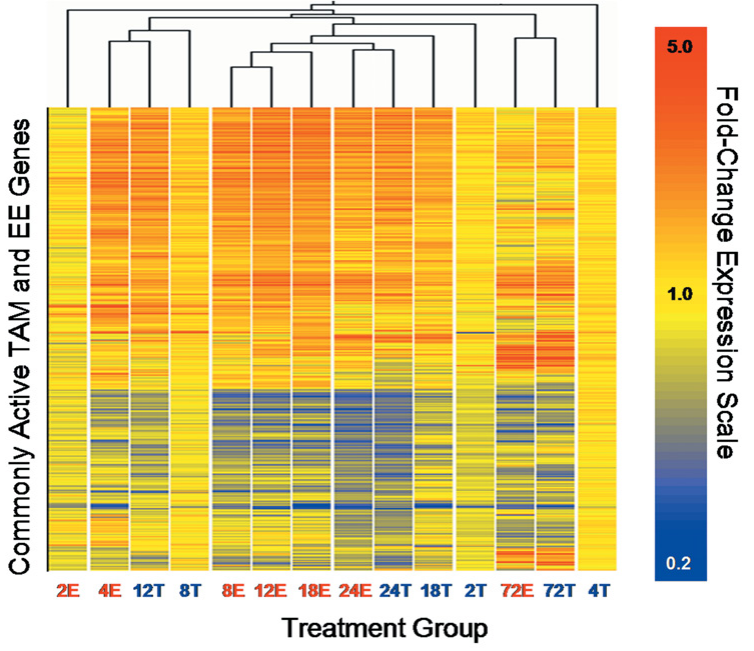
**Temporal comparison of genes commonly activated by TAM and EE**. Hierarchical clustering of 1209 TAM- and EE-regulated genes (y-axis) identifies subsets of similar profiles according to time and treatment (x-axis). The dendrogram indicates that early responses (4 hrs) to ethynylestradiol (E) are most similar to 8 and 12 hrs tamoxifen (T) responses demonstrating temporally displaced TAM activation consistent with the delayed absorption of TAM. However, temporal displacement of TAM elicited responses is not maintained as EE and TAM responses cluster together at 24 and 72 hrs.

Temporal TAM data were compared to an analogous EE study using the same immature, ovariectomized C57BL/6 mouse model [16]. Employing the P1(*t*) > 0.999 and |fold change| ≥ 1.5 criteria, 2657 unique annotated genes were differentially expressed following treatment with 100 μg/kg EE, of which 1209 were also activated by TAM (Additional file 3). Agglomerative hierarchical clustering of common genes by treatment and time indicates that the 12 hr TAM response is most similar to the 4 hr EE response, followed closely by 8 hr TAM (Figure 6). Interestingly, TAM and EE exhibit similar gene expression profiles at 24 and 72 hrs, suggesting that the delay in some TAM-elicited responses is not maintained at later time points.

Expression profiles were compared for the 1209 differentially expressed genes that were regulated by TAM and EE. These genes were categorized as Similar, more Efficacious by EE or TAM, or Ambiguous (Table 2). A total of 793 genes (66%) exhibited expression profiles that were similar in pattern and efficacy when a temporal shift, due to delayed TAM response, was considered. Interestingly, 28 genes that were differentially expressed at least 2-fold more by EE when compared to TAM (i.e., EE Efficacious genes) were associated with cell growth, regulation of transcription and protein metabolism and transport including *Fos *(6.4-fold by EE; 4.1-fold by TAM) and *Inhbb *(7.6-fold by EE; 3.2-fold by TAM). These genes are involved in cell cycle regulation and cellular growth, respectively, and possibly support the greater physiological effect exhibited by EE. In contrast, 19 genes were modulated 2-fold or greater by TAM, including *Sfn *(3.6-fold by EE; 5.5-fold by TAM), which is associated with proliferation inhibition. In general, efficacious TAM elicited responses were associated with receptor-mediated signal transduction, ion transport and protein metabolism.

**Table 2:** Classification of TAM and EE commonly active annotated features

*Classification Category*	*Definition*	*Number of Annotated Genes*
Total Features		1209
Similar (S)	Similar profiles exhibit patterns which are comparable in direction and magnitude across time; this also takes into account temporally shifted responses.	793
EE Efficacious (EEf)	Potent responses demonstrate similar directional responses, but one compound elicits a greater induction or repression, by at least 2-fold, than the other; this category also includes temporally shifted responses.	28
TAM Efficacious (TEf)		19
Ambiguous (A)	Gene pairs which did not fall into the previous four categories were labeled as Ambiguous	369

**Figure 7:**
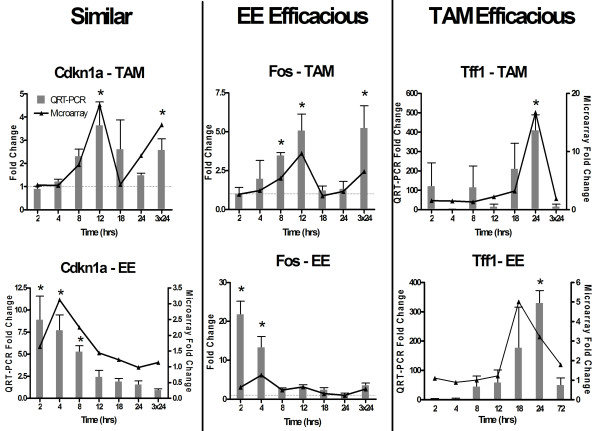
**Examples of TAM and EE differential gene expression classifications**. Examples of representative genes classified as Similar or Efficacious based on microarray data only. QRT-PCR analysis confirmed the classifications of these genes. In some cases (e.g., Cdkn1a) a gene classified as Similar may also be classified as EE-Efficacious based on QRT-PCR results due to data compression inherent in microarray data. Statistically significant differences (p < 0.05, n = 4) due to treatment are denoted by an asterisk (*).

Gene expression comparisons between the two studies were also verified by QRT-PCR. As previously reported, gene expression data is subject to compression [23], and therefore the sensitivity of QRT-PCR data is often greater when compared to microarray data. Thus, some genes classified as Similar may also be classified as EE- or TAM-Efficacious. For example, microarray data suggested that Cdkn1a response to TAM and EE were comparable, but through QRT-PCR EE induced an 8-fold response compared to a 3.5-fold induction by TAM (Figure 7).

TAM and EE responsive genes were also examined for estrogen response elements (EREs) in their promoter regions by comparison to a list of computationally identified sequences [24]. EREs were found in 176 TAM-active genes and 218 EE-active genes, with 133 regulated by both compounds. Only 10% of TAM or EE differentially expressed genes possessed an ERE suggesting that other *trans-*acting factors may also be involved or that EREs were outside of the search regions. Annotation information in public repositories is constantly evolving, thus gene names may have changed or new genes may have been added since the publication. As a result, some genes may be misclassified regarding their ERE status.

### TAM- and EE-specific gene expression data

**Figure 8:**
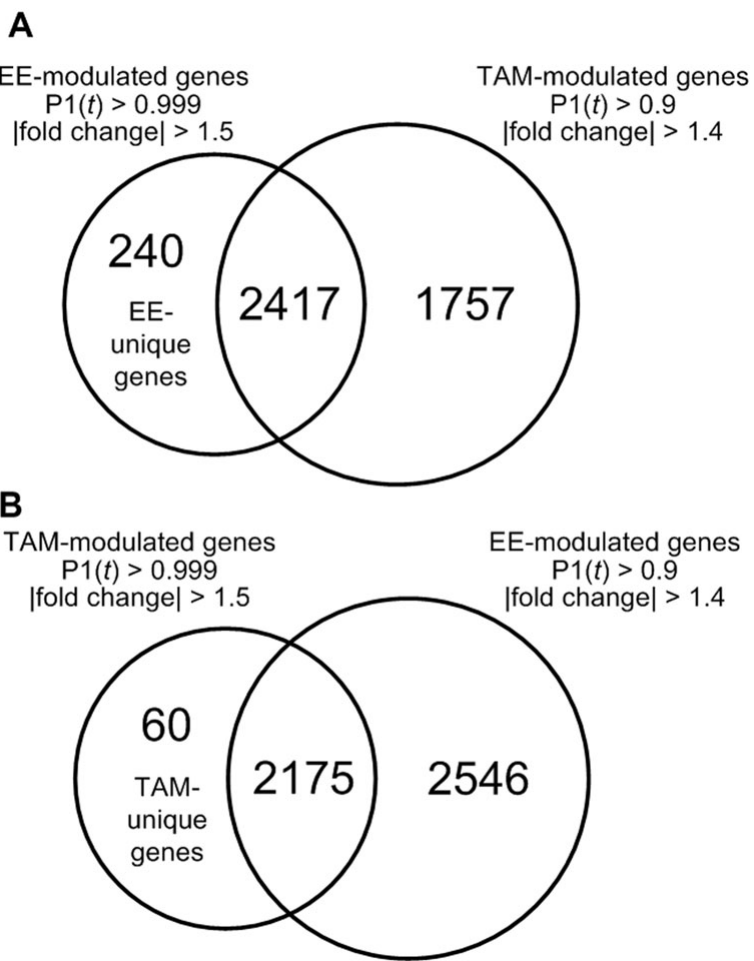
**Identification of unique EE and TAM differentially expressed genes**. Treatment specific differentially expressed genes were identified by excluding a list obtained using a more relaxed criteria (P1(*t*) > 0.9; |fold change| ≥ 1.4) for one treatment from the differentially expressed genes identified using the standard criteria (P1(*t*) > 0.999; |fold change| ≥ 1.5) of the second treatment to identify gene expression changes that were more likely to be unique to one treatment. (A) A liberal list of TAM-induced genes identified, using a relaxed criteria of P1(*t*) ≥ 0.9 and |fold change| ≥ ± 1.4, was excluded from the EE differentially expressed gene list using the standard selection criteria of P1(*t*) ≥ 0.999 and |fold change| ≥ ± 1.5 to identify 240 genes more likely to be differentially expressed by EE alone. (B) Using a similar approach, a list of 60 genes more likely to be differentially expressed by TAM alone was generated. Lists of EE and TAM specific genes are provided in Additional files 4 and 5.

**Figure 9:**
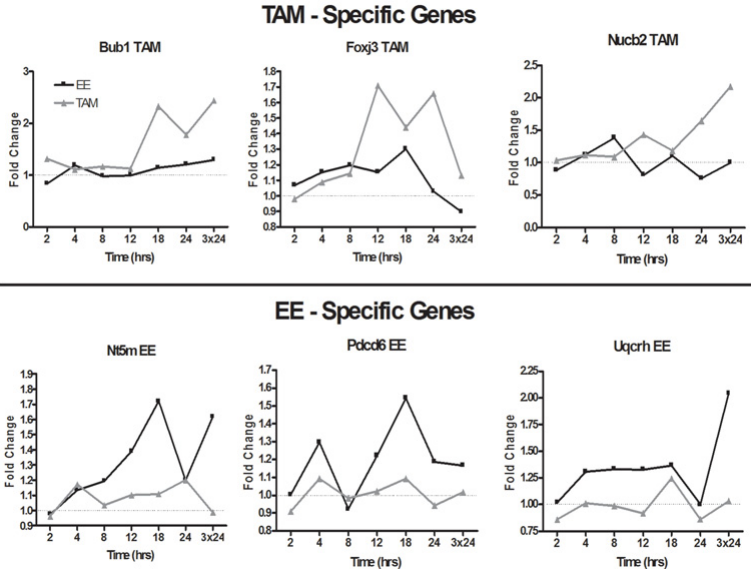
**Temporal expression profiles of TAM and EE-specific genes**. Graphical representation of genes exhibiting compound-specific responses demonstrated profiles which were distinctly different in pattern and magnitude compared to its non-responsive counterpart. These examples further illustrate that the filtering conditions used were adequate to identify differential responses by TAM and EE.

Gene expression changes unique to either TAM or EE may be another factor contributing to their different uterotrophic responses. An additional filtering method was used to identify genes more likely to be unique to EE treatment which involved excluding an extended list of TAM-regulated genes obtained by relaxing the TAM criteria to P1(*t*) > 0.9 and |fold change| ≥ 1.4 from the standard criteria (P1(*t*) > 0.999; |fold change| ≥ 1.5) of EE (Figure 8A). The same approach was also used to obtain a list of genes unique to TAM (Figure 8B). This ensures that those genes significant in both treatments and approaching significance in the other treatment are not considered as unique, thus increasing the likelihood of identifying treatment-specific differential gene expression responses. For example, to identify unique EE responses, the 2417 differentially expressed TAM genes that satisfy the P1(*t*) > 0.9 and |fold change| > 1.4 were excluded from the 2657 differentially expressed EE genes (P1(*t*) > 0.999; |fold change| ≥ 1.5) to identify 240 genes unique to EE treatment (Fig. 8a; Additional file 4). Similarly, genes more likely unique to TAM were identified by excluding the 2175 differentially expressed EE genes with a P1(*t*) > 0.9 and |fold change| > 1.4 that were in common with the 2235 differentially expressed TAM genes (P1(*t*) > 0.999; |fold change| = 1.5) to identify 60 genes more likely unique to TAM (Additional file 5). Treatment-specific responses exhibited profiles distinctly different in pattern and magnitude from their counterpart (Figure 9) even when taking delays, due to TAM, into consideration.

The pathways represented within unique EE-responsive genes include apoptosis regulators (*Bok *and *Pdcd6*) and water imbibition (*Aqp8 *and *Slc22a7*), consistent with the physiological effects observed. Fewer unique TAM-responsive genes were identified. There was not an overrepresentation of any functional pathway consistent with its weaker uterotrophic response. These data suggest that differentially regulate subsets of genes exist that contribute to the distinctive uterotrophic response elicited by each treatment.

## Discussion

A comparative approach was used that integrates the gross organ, histopathological, and morphometric uterine effects of EE and TAM with their dose response and temporal gene expression profiles to further elucidate the molecular basis of the partial agonist activity of TAM. TAM treatment induces a 5-fold increase in gross uterine weight following three daily doses compared to an 11-fold increase with EE. In addition, no significant water imbibition was induced by TAM. These effects are well documented and are the basis for the classification of TAM as a partial agonist [6,19,20,25]. Moreover, TAM induces a delayed increase in uterine weight when compared to EE which may be partially attributed to its weaker agonist activity but is more likely a reflection of slower absorption [26-28]. In contrast, peak serum levels of EE are detected within two hours of treatment [29].

At equi-efficacious doses of TAM and EE (i.e. 100 vs. 20 μg/kg, respectively), comparable effects on UWW, luminal circumference and glandular epithelial were observed (data not shown), suggesting both treatments proceed through similar changes to achieve uterotrophy. However, at higher doses, TAM does not elicit a comparable gamut of responses as seen with higher doses of EE. Surprisingly, TAM increased luminal epithelial thickness [18], due to cellular hypertrophy and hyperplasia, that was not significantly different from EE, but mediated a smaller increase in luminal circumference with more endometrial glands compared to EE. Although these results appear contradictory, glandular epithelium may arise from the luminal epithelium and appear as highly invaginated regions of the lumen that generate a large secretory surface area [30]. Thus, despite fewer endometrial glands in EE samples, its glandular area is greater due to the increased luminal glandular surface area which was not observed in the TAM treated samples.

Temporal tamoxifen-elicited gene expression profiles were examined following a single dose as well as after three daily doses of 100 μg/kg TAM. Only 9 features, representing 6 annotated genes, exhibited differential expression at 2 and 4 hrs after TAM treatment compared to 1234 EE genes at the same time points [16], consistent with the delayed histological effects. Of these early TAM responses, only Esr1 and Car3 have been reported to be induced by estrogen [16,31]. At 12 hrs, 683 genes were differentially expressed in response to TAM, of which 541 genes were also affected by EE between 2 and 8 hrs [16]. Agglomerative hierarchical clustering suggests that genes affected by TAM and EE exhibited comparable gene expression changes despite the delay in TAM responses.

Genes regulated by TAM and EE represent a variety of pathways including cell cycle regulation, cytoskeletal re-organization, nucleotide metabolism, immune and complement activation and lipid transport and metabolism, and have previously been associated with eliciting the uterotrophic response [15,16,22,32-34]. Similarities in their gene expression profiles suggest that the uterotrophic response involves a defined subset of genes mediated by the ER. Furthermore, greater than 75% of TAM-activated genes that possessed an ERE, were also activated by EE. However, differences in efficacy and responsive genes may partially explain uterotrophic response differences.

Despite temporal delays, many genes were regulated by both EE and TAM. Most of these commonly active genes exhibited comparable fold changes suggesting that they do not significantly influence the magnitude of the uterotrophic response. For instance, both treatments equally repressed uterotrophic supportive pro-apoptotic caspases (*Casp2 *and *Casp6*) (reviewed in [35]). Although these genes were responsive to EE and TAM, others demonstrated quantitative differences in their expression behavior. Twenty-eight genes, including the proliferation supportive genes *Cdkn1a*, *Fos *and *Inhbb*, exhibited greater EE efficacy consistent with their previously reported estrogen-induced expression [36-38] resulting in a full uterotrophic agonist response. In contrast, 22 genes more highly induced by TAM included G2/M inhibitor (*Sfn/14-3-3σ*), which has been associated with human endometrial carcinomas [39] to reduce proliferation. Many of these quantitative differences in gene expression efficacy are consistent with the potent agonist activity of EE and the weak agonist activity of TAM.

There were also treatment-specific gene expression effects. Tentatively, 240 and 60 modulated genes were identified as unique to EE or TAM, respectively. In general, these responses were consistent with uterotrophic activity elicited by EE and TAM. For example, QRT-PCR verified the early induction of mitotic gene, *Anapc1 *by EE (data not shown). Also, the treatment specific repression of pro-apoptotic Bcl-2 member, *Bok*, and the induction of *Pdcd6*, an apoptosis regulator, associated with proliferating tissues [40] are consistent with the greater efficacy of EE. *Bok *has previously been shown to be EE responsive in uteri, whereas *Pdcd6 *approached the statistical cut-off in a previous study [15]. For TAM, QRT-PCR confirmed decreased expression of *Sipa1 *(data not shown), a repressed response at 24 hrs associated with decreased proliferation [41] that may reduce hyperplasia.

DNA synthesis and replication pathways were also differentially regulated. Sustained up-regulation of dNDP phosphorylating genes, *Nme1 *and *Nme6 *[42], suggest salvage pathways are emphasized for nucleotide synthesis rather than *de novo *processes where *Prps1*, the first step in purine biosynthesis, is repressed during the same period. These genes are similarly modulated by TAM and EE suggesting that proliferation may deplete resources for *de novo *synthesis. Only *Nme1 *has been previously shown to be EE responsive in rodent uteri [15,16]. However, EE uniquely inhibited the *de novo *pyrimidine synthesis gene, *Dhodh *[18–72 hrs], and induced the nucleotide recycling gene, *Nt5m *[18 and 72 hrs] [43] suggesting an involvement of salvage pathways to support EE-induced proliferation which have not previously been reported to be estrogen responsive.

Water imbibition is a characteristic uterine response to estrogens involving the increased flow of water to the lumen mediated by aquaporins and ion transporters [44]. It does not appear to be a factor in TAM-induced uterine weight increases as blotted weights were not significantly different from wet weights. *Aqp1 *and *Aqp5 *are comparably regulated by TAM and EE, while *Aqp8 *induction was specific to EE (QRT-PCR verified, data not shown). *Aqp8 *is a known contributor to water imbibition [45] and its EE-specific response suggests it may play a larger role in the process of a full uterotrophic response.

The lack of ion transporter regulation may also be a contributing factor in the absence of TAM-induced water imbibition. The EE induction of zinc transporter, *Slc30a3 *[12 hrs], which causes ion uptake into various vesicle compartments [46,47] may facilitate stromal edema and has been shown to be responsive to estrogen where it is down-regulated in brain tissue [48]. Organic anion transporter, *Slc22a7*, was repressed by EE from 18 – 72 hrs in the uteri suggesting anion retention in the stroma that may also be important for edema. *Slc22a7 *is an importer in the basolateral membrane of kidney tubule epithelia (reviewed in [49]), and is estrogen responsive in the kidney [50].

Differential regulation of ATP production genes is also consistent with the greater uterotrophic efficacy of EE. Transcripts associated with oxidative phosphorylation (OXPHOS) complex I, *Ndufb8 *[8–24 hrs], and complex III, *Uqcr *[8–18 hrs] and *Uqcrh *[4–18, 72 hrs], were all up-regulated. Although not previously been reported as responsive, collectively, the EE modulation of OXPHOS components is consistent with greater energy demands required to support increasing hypertrophic and hyperplastic activity induced by EE compared to TAM.

Other TAM gene expression studies have been conducted using *in vitro *breast cancer models, primarily MCF-7 cells. Comparisons of differentially expressed gene lists identified minimal to no overlap of TAM responses between *in vitro *human breast tissue and *in vivo *mouse uterus [51,52]. Only the induction of *Uqcrb *[53], *Nqo1 *[54], *Tff1*, *Mapt *[55], *Pctk3*, *Wnt4 *[56], *Myb*, *Cdc6*, *Cdc20*, *Mcm2*, *Fos *and *Mybl2 *[57] and repression of *Xrcc1*, *Tgfa *[54], *Rap1ga1*, *Blnk*, *Tm4sf1*, *Matn2*, *Ifi30*, *Tgfb3 *and *Smpd1 *[55] correlated with the changes observed in the current study. Moreover, there are examples of divergent gene expression changes such as inverse responses for *Pfn2 *[54], *Ctsh*, *Selenbp1*, *Nfrkb*, *Cyp1a1 *[55], *Prps1 *and *Tmsb4x *[56]. The long term uterine effects of TAM have also been examined in mice following neonatal exposure. Mice were treated for four consecutive days after treatment and uteri samples examined at various months after dosing [58]. *Col1a1 *exhibited persistent up-regulation months after treatment and was also induced in our short term study. Several factors, in addition to model differences, likely contribute to the minimal overlap including differences in array platforms and genome coverage, study design, and data analysis. For example, E2 and 4OH-TAM were utilized in the *in vitro *studies while EE and TAM were administered to the mice.

Despite the minimal overlap between the models, the activities of TAM, when compared to E2 were comparable. *In vitro *and *in vivo*, the gene expression changes elicited by 4OH-TAM were similar to those mediated by E2 in MCF-7 cells. Furthermore, the magnitude of gene expression changes due to 4OH-TAM was attenuated compared to E2 [55,57]. Although 4OH-TAM and EE induced similar cell cycle genes, other down-stream mechanisms were also regulated to prevent 4OH-TAM mediated cell cycle progression [57]. Some of these mechanisms may play a roll in the partial uterotrophic response elicited by TAM in treated mice.

Differences in chemical structure may also contribute to ligand specific responses. TAM belongs to the stilbene/triphenylethylene family while EE is steroidal. Each has unique binding modes resulting in different ER conformations [3], binding affinities [59,60], ligand-induced binding domain topographies [61], coactivator recruitment capabilities [62,63], gene-specific thresholds of activation, and efficacies [64]. Specifically, 4OH-TAM induces a different conformational change in the ER compared to E2, influencing interactions with different coactivators. Electrophoretic mobility shift assay and crystallographic examination [65] have shown that 4OH-TAM-bound ER could not bind a GRIP1 coactivator LXXLL peptide due to helix-12 interference at the binding cleft, which was recruited by E2. Consequently coactivator recruitment may influence receptor complex interactions with response element variants [66] which has been shown with other structurally diverse ligands and nuclear receptors [67,68].

In addition, differences in absorption, distribution, metabolism and excretion (ADME) between ligands and species, likely contribute to divergent physiological and gene expression characteristics. It is well documented that TAM metabolism differs significantly between humans and rodents, for example, TAM *N*-oxide, 4OH-TAM and DMT are the predominant metabolites in the mouse, while DMT is the major human metabolite in microsomal studies [28,69,70]. In rodents, the levels and rates of TAM metabolism to 4OH-TAM and DMT were significantly different in the rat and mouse, where the rat metabolite profile more closely resembles human profiles [28].

A cytochrome P450 2D6 polymorphism in humans further illustrates the potential effects of differences in metabolism on TAM activity. 4-OH-*N*-desmethyltamoxifen (endoxifen) is a recently identified TAM metabolite, found at higher levels than 4OH-TAM in patient serum, generated by CYP2D6 activity. It exhibits similar ER binding affinity, and comparable breast cancer cell proliferation and estrogen-induced pS2 mRNA expression inhibition activities compared to 4OH-TAM [4]. However, patients expressing specific CYP2D6 polymorphisms (i.e., CYP2D6*3, *4, *5 and *10) that impaired or abolished CYP2D6 metabolism have a nearly 2-fold higher risk of breast cancer recurrence [71]. Collectively, these studies illustrate the significant differences in TAM metabolism between models that compromise the extrapolation of rodent data for use in human risk assessment.

## Conclusion

Despite the comprehensive time course and dose response studies, a complete assessment of the gene expression effects and their roles in uterine responses could not be achieved due to limited genome coverage on our custom cDNA arrays and incomplete functional annotation for the represented genes. However, comparative TAM and EE studies using comparable designs and models identified conserved functionally annotated gene expression changes that are consistent with the measured uterotrophic response. Qualitatively, TAM and EE gene expression profiles are similar; however, there are quantitative differences in efficacy, consistent with the partial agonist activity of TAM. Despite the evidence for these qualitative and quantitative differences in gene expression, demonstration that these changes have causal roles in the partial uterotrophic response elicited by TAM is required. The relevance of the differences between estrogen and TAM and the association with endometrial cancer [9,72,73] also needs further investigation.

## Methods

### Animal husbandry and treatment

Female C57BL/6 mice, ovariectomized by the vendor on postnatal day (PND) 20, were obtained from Charles River Laboratories (Raleigh, NC) on PND 25. Groups of five mice were housed in polycarbonate cages bedded with cellulose fiber chips (Aspen Chip Laboratory Bedding, Northeastern Products, Warrensberg, NY) in a 23°C environment with 30–40% humidity and a 12 h light/dark cycle (0700 – 1900 h). Animals had access to deionized water and Harlan Teklad 22/5 Rodent Diet 8640 (Madison, WI) *ad libitum *and acclimatized for 4 days prior to treatment. For the dose response study, animals (n = 5) were orally gavaged with 0.1 mL of 1, 3, 10, 30, 100, 300 or 1000 μg/kg b.w. tamoxifen (≥ 99% pure, *trans*-2- [4-(1,2-Diphenyl-1-butenyl)phenoxy]-*N*,*N*-dimethylethylamine) (Sigma Chemicals, St. Louis, MO), 100 μg/kg b.w. 17α-ethynylestradiol (EE; 17*α*-Ethynyl-1,3,5(10)-estratriene-3,17*β*-diol) (Sigma) or sesame oil vehicle (Sigma) alone. Standard uterotrophic regimen was followed [12], consisting of three daily doses followed by sacrifice 24 hrs after the final treatment, (3 × 24 hrs). Doses were prepared based on average animal weight. For the time course study, animals (n = 5) were orally gavaged once or three times daily (3 × 24) with 100 μg/kg b.w. TAM or vehicle alone and sacrificed at 2, 4, 8, 12, 18 and 24 hrs after treatment in addition to 3 × 24 hrs treatment group. Animals were sacrificed by cervical dislocation and animal body weights were recorded. The uterus was transected at the border of the cervix, and stripped of extraneous connective tissue and fat. Whole uterine weights were recorded before (wet weight) and after blotting (blotted weight) under pressure with absorbent tissue. A 6–8 mm section of uterine horn was not blotted and placed in 10% neutral buffered formalin (NBF) for histological preparation while the remainder was snap frozen in liquid nitrogen and stored at -80°C for RNA extraction. All procedures were performed with the approval of the Michigan State University All-University Committee on Animal Use and Care.

### Histological processing, morphometric and pathological analysis

Samples stored in 10% NBF were allowed to fix for at least 24 hrs at room temperature then placed into tissue cassettes and stored in 30% ethanol holding solution at 4°C. Paraffin embedding, 5 μm sectioning, mounting and hematoxylin and eosin staining were completed by the Michigan State University Laboratory for Anatomical Histology and Molecular Sciences according to standard techniques [74]. Pathological assessments were evaluated according to standardized National Toxicology Program (NTP) pathology codes.

Morphometric analysis was performed on midhorn uterine cross sections for all animals (n = 5 per treatment group) using Scion Image analysis software (Scioncorp, Frederick, MD). Histological markers of uterotrophy, including luminal epithelial cell height (LECH), luminal circumference and number of endometrial glands were quantified for each slide. Statistical analysis of morphometric data was assessed by Dunnett's or two-way ANOVA followed with Tukey's HSD *post hoc *analysis to examine dose dependent and temporal effects, respectively (SAS version 9.1).

### RNA isolation

Briefly, 1.0 mL of Trizol (Invitrogen, Carlsbad, CA) was added to the frozen uterine tissue in a 2.0 mL microfuge tube and homogenized in the presence of steel beads by a Mixer Mill 300 homogenizer (Retsch, Germany). Total RNA was isolated and extracted according to the manufacturer's protocol and resuspended in The RNA Storage Solution (Ambion, Austin, TX). RNA samples were quantified spectrophotometrically (A_260_) and assessed for quality by A_260_/A_280 _ratio as well as inspected using denaturing agarose gel electrophoresis.

### Microarray hybridization and analysis

Custom in-house cDNA arrays consisting of 13,361 features, representing 7,952 unique genes (Unigene Build 144), were spotted on epoxy coated glass slides (SCHOTT Nexterion, Germany) using an Omnigrid arrayer (GeneMachines, San Carlos, CA) and Telechem Chipmaker 3 pins in a TeleChem CHP3 printhead head (Telechem International Inc., Sunnyvale, CA) by the Research Technology Support Facility at Michigan State University [75]. Selected clones were obtained from EPAMAC [76], Research Genetics, the National Institute of Aging and Lion Biosciences. Detailed protocols for processing of microarrays are available at [77].

An independent reference study design was used to assess treatment effects [14]. For the dose response study, each treatment group was hybridized to a single vehicle pool utilizing 14 arrays, including dye swaps, and 3 biological replicates for a total of 42 arrays. For the time course study, each time-matched treated and vehicle sample was competitively hybridized utilizing 14 arrays, including dye swaps with 3 biological replicates for a total of 42 arrays. The Genisphere 900 3DNA Array Detection (Genisphere Inc., Hatfield, PA) indirect incorporation kit was used to generate cDNA samples for hybridization. Briefly, 1 μg of RNA was reverse transcribed in the presence of an oligo-tagged primer specifically targeted for Cy3- or Cy5- conjugated dendrimers. The cDNA was resuspended in 58 μL of 2X Formamide-Based Hybridization Buffer and hybridized overnight on arrays sealed in a light-shielded, humid chamber submerged in a 42°C water bath incubation. Slides were then washed in SSC solutions containing decreasing concentrations of SDS, spin-dried and re-hybridized with a Cy3:Cy5 (1:1) dendrimer mixture in formamide based buffer to indirectly incorporate dyes at the Cy3- and Cy5-dendrimer-tagged cDNA hybridized on the first day. Slides were washed and dried as previously described, and scanned at 635 nm (Cy3) and 532 nm (Cy5) using a 428 Affymetrix Scanner (Santa Clara, CA). Images were examined, features identified and intensity values recorded using GenePix v.5.1 (Molecular Devices).

### Microarray quality control, statistical analysis and gene list filtering

All arrays in this study were compared to a historical data set of high quality arrays. Parameters assessed included background signal intensity, feature signal intensity, feature vs. background signal intensity ratios, the number of features with background intensities greater than the feature intensity for each array, and relationships between feature and background signal intensities. All arrays surpassed the quality control parameters established in this laboratory [78].

Data were normalized using a semi-parametric approach [79] and model-based *t*-values were calculated comparing time-matched treated and vehicle samples. Posterior probabilities of activity [P1(*t*)-value] were then calculated on a per-gene and per-time point basis using an Empirical Bayes analysis [80]. Gene lists were initially filtered based on posterior probability (P1(*t*) > 0.999) and fold-change cut-off (|fold change| > ± 1.5) resulting in an active gene list on which further functional analysis was conducted. All raw and analyzed data were stored in dbZach [77], a Minimum Information About Microarray Experiments (MIAME)-supportive relational database [81] running under Linux/Oracle 10 g. dbZach currently supports microarray data storage, retrieval, and querying as well as facilitates data analysis, sharing and reporting [82].

Active gene lists exclusive to TAM and EE were also generated. Data for the EE time course has previous been published [16]. The TAM unique gene list was generated based on relaxed criteria (P1(*t*) > 0.9 and |fold change| > ± 1.4 cut-off) to obtain a liberal EE-mediated gene list which was then excluded from the original TAM unique gene list using P1(*t*) > 0.999 and |fold change| > ± 1.5 criteria. The EE unique gene list was generated using a reciprocal approach (i.e., relaxed criteria (P1(*t*) > 0.9 and |fold change| > ± 1.4 cut-off) to obtain a liberal TAM-mediated gene list which was then excluded from the original EE unique gene list using P1(*t*) > 0.999, and |fold change| > ± 1.5 criteria). This approach ensured that genes marginally missing the cut-offs were not included in the compound-unique list.

Estrogen response element searches were completed by comparing Gene Symbols to the computationally identified list compiled by Bourdeau et al. [24].

### QRT-PCR

Aliquots of RNA isolated from each of the five replicates were set aside for SYBR™ Green quantitative real-time PCR (QRT-PCR) verification. EE-treated, temporal mouse uteri RNA were previously isolated [16]. An oligo-dT anchored Superscript II (Invitrogen) reverse transcriptase reaction was carried out on 1 μg of RNA, in a 20 μL reaction, from each biological sample as per manufacturer's instructions. Samples were diluted four-fold and 3 μL used in a 30 μL real-time reaction mix containing 1X SYBR Green PCR buffer, 3 mM MgCl_2_, 0.33 mM dNTPs, 0.5 IU AmpliTaq Gold (Applied Biosystems, Foster City, CA) and 0.15 mM forward and reverse primer. All primers were designed by submitting cDNA microarray clone sequences into Primer3 [83] to obtain an amplicon of approximately 125 bp (Additional file 6). PCR amplification was conducted in 96-well MicroAmp Optical plates (Applied Biosystems) on an Applied Biosystems PRISM 7000 Sequence Detection System under the following conditions: 10 min denaturation and enzyme activation at 95°C, followed by 40 cycles of 95°C for 15 s and 60°C for 1 min. After amplification, a 30 min dissociation protocol was conducted to assess primer specificity and product uniformity. Each plate contained duplicate standards of purified PCR product of known template concentration over eight orders of magnitude to generate a log template concentration standard curve. No template controls (NTC) samples were included on each plate such that experimental samples within 2 standard deviations of the NTCs are considered below the limits of detection. Plots were visualized and thresholds determined using ABI Prism 7000 SDS Software (Applied Biosystems). Results were normalized to a geometric mean of beta-actin (Actb), glyceraldehydes-6-phosphate dehydrogenase (Gapd) and hypoxanthine guanine phosphoribosyl transferase (Hprt) mRNA levels to control for differences in RNA loading, quality and cDNA synthesis. Statistical significance of expression differences between vehicle and TAM treated samples were assessed by two-way ANOVA followed by Tukey's HSD *post hoc *analysis to examine treatment and treatment over time effects (SAS version 9.1). Correlation analyses of QRT-PCR and microarray data generated using the correlation function of R v2.1.0.

### Immunohistochemistry

Rabbit polyclonal antibodies specific for PCNA were purchased from Abcam, Inc. (Cambridge, MA) and staining localized using manufacturer's instructions for the Vectastain Elite ABC Kit (Vector Laboratories, Burlingame, CA). Briefly, paraffin-embedded uterine sections were placed on glass slides, deparaffinized in xylene and re-hydrated through a series of decreasing ethanol concentration washes ending in ddH_2_O. Endogenous peroxidases were quenched in 0.3% H_2_O_2 _in methanol solution (30 min) followed by boiling (15 min) in a 10 nM sodium citrate solution (pH 6.0) for antigen retrieval. To minimize nonspecific background staining, sections were blocked with normal goat serum (Vector Laboratories) for 20 min. The slides were incubated for 1 hr with the primary rabbit anti-PCNA polyclonal antibody (1:500 dilution in PBS), followed by 30 min each with biotinylated goat anti-rabbit antibody (Vector Laboratories) (1:400) and ABC reagent (Vector Laboratories). A single PBS rinse was performed between incubations with each antibody. Localization of antigen was obtained using Vector^® ^NovaRED (Vector Laboratories). The sections were counterstained with hematoxylin.

## Authors' contributions

CJF organized the studies, performed all microarray and immunohistochemistry assays, conducted and statistically analyzed QRT-PCR assays, as well as compiled and interpreted the data collected and generated the primary draft manuscript. LDB provided database support for the microarray data including quality control assessments and statistical. KJW conducted all the pathological assessments of the histological slides prepared. ALF prepared samples for histological slide preparation, collected morphometric of histological slides and conducted statistical analyses of the data. TRZ conceived the study and its design and supervised its completion. All authors read and approved the final draft of the manuscript.

## Supplementary Material

Additional file 1 Tamoxifen treated time course data. Click here for file

Additional file 2 Tamoxifen treated dose response data. Click here for file

Additional file 3 List of time course clones active for both EE and TAM treatments. Click here for file

Additional file 4 List of active time course clones unique to ethynyl estradiol treatment. Click here for file

Additional file 5 List of active time course clones unique to tamoxifen treatment. Click here for file

Additional file 6 QRT-PCR primer list. Click here for file
